# Vδ2+ T cell response to malaria correlates with protection from infection but is attenuated with repeated exposure

**DOI:** 10.1038/s41598-017-10624-3

**Published:** 2017-09-13

**Authors:** Prasanna Jagannathan, Fredrick Lutwama, Michelle J. Boyle, Felistas Nankya, Lila A. Farrington, Tara I. McIntyre, Katherine Bowen, Kate Naluwu, Mayimuna Nalubega, Kenneth Musinguzi, Esther Sikyomu, Rachel Budker, Agaba Katureebe, John Rek, Bryan Greenhouse, Grant Dorsey, Moses R. Kamya, Margaret E. Feeney

**Affiliations:** 1 Department of Medicine, Stanford University, Stanford, CA USA; 2 Department of Medicine, University of California San Francisco, San Francisco, CA USA; 3 Infectious Diseases Institute, Kampala, Uganda; 4 Makerere University College of Health Sciences, Kampala, Uganda; 5 Burnet Institute, Disease Elimination (Malaria), Melbourne, Australia; 6 Infectious Diseases Research Collaboration, Kampala, Uganda; 7 Department of Pediatrics, University of California San Francisco, San Francisco, CA USA

## Abstract

Vδ2^+^ γδ T cells are semi-innate T cells that expand markedly following *P. falciparum (Pf)* infection in naïve adults, but are lost and become dysfunctional among children repeatedly exposed to malaria. The role of these cells in mediating clinical immunity (i.e. protection against symptoms) to malaria remains unclear. We measured Vδ2^+^ T cell absolute counts at acute and convalescent malaria timepoints (n = 43), and Vδ2^+^ counts, cellular phenotype, and cytokine production following *in vitro* stimulation at asymptomatic visits (n = 377), among children aged 6 months to 10 years living in Uganda. Increasing age was associated with diminished *in vivo* expansion following malaria, and lower Vδ2 absolute counts overall, among children living in a high transmission setting. Microscopic parasitemia and expression of the immunoregulatory markers Tim-3 and CD57 were associated with diminished Vδ2^+^ T cell pro-inflammatory cytokine production. Higher Vδ2 pro-inflammatory cytokine production was associated with protection from subsequent *Pf* infection, but also with an increased odds of symptoms once infected. Vδ2^+^ T cells may play a role in preventing malaria infection in children living in endemic settings; progressive loss and dysfunction of these cells may represent a disease tolerance mechanism that contributes to the development of clinical immunity to malaria.

## Introduction

Despite declines in malaria morbidity in parts of sub-Saharan Africa^1^, malaria causes hundreds of thousands of deaths annually, predominantly among young children^1, 2^. Children residing in endemic areas eventually acquire ‘clinical’ immunity to malaria (i.e. they are protected against symptoms)^3–5^, but they commonly harbor parasites as asymptomatic and transmitting carriers^6, 7^. Although individuals generally do not appear to develop sterilizing immunity that prevents any infection, blood-stage parasite density declines with age and repeated exposure^8^, suggesting the development of immune responses that are able to limit blood stage replication. Importantly, pro-inflammatory responses that limit parasitemia may also lead to clinical symptoms; thus, ‘clinical’ immunity could depend upon the ability to down-modulate such responses, as suggested by recent data from our group and others^9–11^.

The Vγ9 Vδ2 subset of γδ T cells, which constitute 0.5 to 5% of peripheral T cells in humans, have been shown to robustly proliferate and produce pro-inflammatory cytokines in response to *Pf* antigen stimulation and to markedly expand following malaria infection in naïve hosts^12–17^. These cells (hereafter termed Vδ2 T cells) rapidly react to phosphoantigens produced by the plasmodial apicoplast, and have been shown to inhibit parasite growth *in vitro* via the release of cytotoxic granules containing granulysin^18, 19^. Given these attributes, Vδ2 T cells may function as ready-made effector cells, and may be most important early in response to malaria infection, potentially before the adaptive immune response to *Pf* has developed. Supporting this hypothesis, cytokine production from these cells has been associated with protection from high density *P falciparum* infection^20^, and higher baseline percentages of these cells have recently been associated with protection from subsequent *Pf* infection among individuals receiving an experimental attenuated sporozoite vaccine^21^.

While Vδ2 T cells may play role in limiting parasite replication, their production of pro-inflammatory cytokine has been implicated in the pathogenesis of severe symptoms from malaria^22^. Thus, curtailing excessive Vδ2 T cell activation may be required for the development of clinical immunity to malaria. We have previously shown that repeated malaria was associated with a loss of Vδ2^+^ T cells in peripheral blood, decreased proliferation and cytokine production of these cells in response to malaria antigen stimulation, and upregulation of numerous genes associated with dampening of the immune response^9, 23^. Furthermore, loss and dysfunction of Vδ2^+^ T cells was associated with a lower likelihood of symptoms upon subsequent infection^9^. Notably, we did not find a significant association between Vδ2^+^ T cell parameters and protection from subsequent infection, although our prior studies were limited to small cohorts of children <5 years of age and were unable to fully account for heterogeneous exposure to mosquitoes.

In the present study, we extend our prior observations regarding the potential role of Vδ2^+^ T cells in mediating clinical immunity to malaria, leveraging large and comprehensively characterized cohorts of children age 6 months to 10 years from two regions of Eastern Uganda with differing transmission intensities [17]. We first evaluated Vδ2^+^ T cell absolute counts following symptomatic malaria episodes, hypothesizing that older children – who have sustained more cumulative malaria exposure in a high transmission setting – would exhibit diminished *in vivo* Vδ2^+^ T cell proliferation. We then evaluated Vδ2^+^ T cell absolute counts, cellular phenotype and *Pf* stimulation-induced IFNγ and TNFα-production from asymptomatic children living in both high and low transmission settings, assessing relationships between these parameters with age, parasitemia, and malaria infection. Finally, we analyzed the relationship between Vδ2^+^ T cell parameters and prospective protection from both *Pf* infection and the likelihood of symptoms once infected. We adjusted our analyses for heterogeneity in exposure to mosquitos using household-level mosquito capture data [18,19]. We hypothesized that higher Vδ2^+^ T cell numbers and cytokine production would be associated with protection from infection, but that greater cytokine production from these cells would also be associated with symptoms among children who are infected.

## Results

### Symptomatic malaria is followed by *in vivo* expansion of Vδ2^+^ T cells in young but not in older children

**Figure 1:**
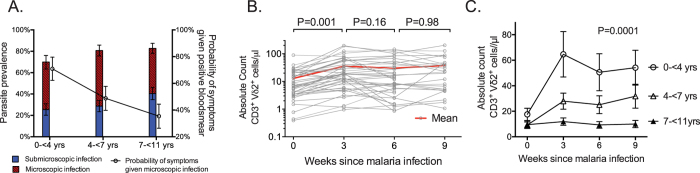
Age associated differences in probability of symptoms if infected and malaria-associated *in vivo* Vδ2^+^ T cell expansion among children in high transmission setting. (**A**) Increasing parasite prevalence, but lower probability of symptoms given patent infection with increasing age among children in Nagongera, Uganda. Point estimates and 95% confidence intervals derived using multilevel mixed effects logistic regression modeling. (**B**) Absolute Vδ2 T cell counts at time of malaria and 3, 6, and 9 weeks post-malaria (n = 43, Wilcoxon matched pairs signed-rank). (**C**) Absolute Vδ2 T cell counts during and after acute malaria episode, stratified by age (0- < 4 yrs, n = 13; 4- < 7 yrs, n = 16; 7–11 yrs, n = 14). Point estimates derived from repeated measures analysis using generalized estimating equations, controlling for Day 0 parasite density.

It has previously been shown that both the absolute count and percentage of Vδ2^+^ T cells expand following a symptomatic malaria infection in naïve and malaria-susceptible adults^15, 24^. Thus it is somewhat paradoxical that we recently found Vδ2^+^ T cell frequencies to be markedly *lower* among two cohorts of Ugandan children following chronic and repeated malaria exposure^9, 23^. To address these seemingly contradictory observations, we measured absolute Vδ2^+^Vγ9^+^ CD3^+^ cell counts at the time of an acute malaria episode and 3, 6 and 9 weeks post-infection among children aged 6 months to 10 years living in a highly endemic region of Uganda. In this setting, parasite prevalence (including both microscopic and sub-microscopic parasitemia) is very high, but the probability of symptoms if blood smear positive decreases dramatically with increasing age (Fig. 1a), consistent with the development of clinical immunity to malaria. Overall, we observed significant expansion of Vδ2^+^Vγ9^+^ T cell counts three weeks after an acute malaria episode (P = 0.001), with stable counts thereafter, although there was significant heterogeneity among individuals (Fig. 1b.) When stratified by age (<4 yrs, 4–7 yrs, and >7yrs), children aged 0− < 4 years had significantly greater Vδ2^+^ T cell expansion following acute malaria than children in older age strata (P < 0.001, Fig. 1c). Indeed, children aged >7 years had no significant expansion of Vδ2^+^ T cells, suggesting that expansion may be blunted following chronic repeated exposure. In contrast, total lymphocyte counts increased following acute malaria but this expansion was not influenced by age (P = 0.24). Together, these data indicate that there are significant age-associated differences in expansion of Vδ2^+^ T cells following acute malaria in endemic settings, with younger children having significantly greater expansion than older children.

### Increasing age is associated with lower absolute counts of Vδ2^+^ T cells in high, but not low, transmission settings

**Figure 2:**
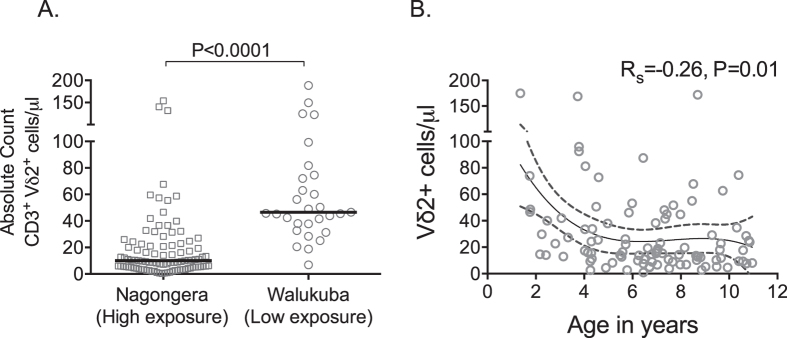
Vδ2^+^ T cell counts decline with increasing age among children living in high transmission setting. (**A**) Absolute CD3^+^Vδ2^+^ cells/μl among asymptomatic children aged 6 months to 11 years in high (Nagongera) vs low (Walukuba) transmission setting at the time of routine assesments, Wilcoxon Rank Sum. (**B)** Absolute CD3^+^ Vδ2^+^ cells/μl by age in Nagongera. R_s_: Spearman *Rho*. Solid line represents best fit regression line and dashed line represents 95% CI.

We have previously shown that repeated malaria leads to decreased percentages of circulating Vδ2^+^ T cells among children ≤4 years of age^9, 23^, but it is not clear whether this is due to an absolute vs. relative loss, as total lymphocyte counts decline gradually during childhood^25^. We assessed the absolute count of Vδ2^+^ T cells/μl among children aged 1–11 years of age living in high prevalence Nagongera and compared these with age-matched children living in the lower malaria transmission setting of Walukuba, Jinja. Children living in Nagongera had significantly fewer Vδ2^+^ T cells/μl (Fig. 2a), as well as a lower percentage of Vδ2^+^ T cells among total CD3^+^ cells (Supplementary Fig. 2a) than those living in Walukuba. In contrast, absolute total T cell counts (CD3^+^ lymphocytes) were similar in the two settings (Supplementary Fig. 1b), suggesting that malaria exposure leads to a selective loss of Vδ2^+^ T cells.

Some studies performed in non-malaria endemic settings have observed an age-associated increase in Vδ2^+^ T cells during childhood^26, 27^. We observed a sharp decline in Vδ2^+^ T cells between 0 to 4 years of age, plateauing between 4 and 11 years of age, among children living in Nagongera (Fig. 2b, Supplemental Fig. 2b), and no significant age-associated change in absolute Vδ2^+^ T cell counts among children in Walukuba, the lower transmission setting (Rs = −0.21, P = 0.24). Furthermore, we observed a subtle, but statistically significant, inverse association between age and Vδ2^+^ T cell production of the inflammatory cytokines IFNγ and TNF following *in vitro* stimulation with P. *falciparum*–infected RBCs among children living in Nagongera (Rs = −0.15, P = 0.02), but not in the lower transmission setting (Rs = 0.09, P = 0.65). These data suggest that increasing age – a surrogate for cumulative exposure to malaria in high transmission settings - is associated with loss, in both percentage and absolute count, and dysfunction of circulating Vδ2^+^ T cells among children living in a high transmission setting.

### Microscopic, but not submicroscopic, parasitemia is associated with lower Vδ2^+^ T cell counts and cytokine production following *in vitro* stimulation

**Figure 3:**
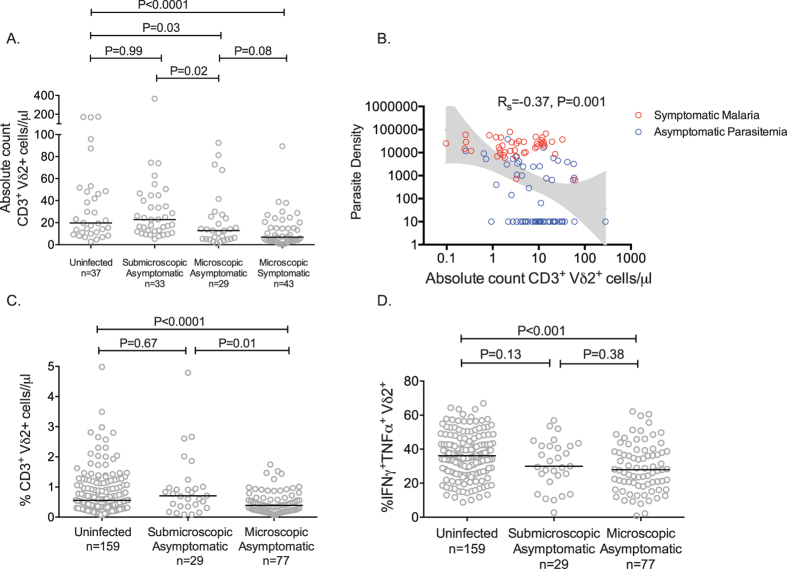
Concurrent microscopic parasitemia associated with diminished Vδ2^+^ T cell counts and cytokine production following *in vitro* stimulation. (**A**) Absolute CD3^+^ Vδ2^+^ cells/μl among uninfected, submicroscopically infected, microscopically infected/asymptomatic, and microscopically infected/symptomatic children living in high transmission setting. (**B**) Association between absolute CD3^+^ Vδ2^+^ cells/μl and parasite densities among infected children (submicroscopically infected children have parasite density estimated at 10 parasites/μl). Shown is 95% confidence interval of best fit regression line. (**C**) Percentage of Vδ2^+^ cells among total CD3^+^ and (**D**) Percentage of Vδ2^+^ T cells producing cytokines following *in vitro* stimulation with *P. Falciparum* infected red blood cells among uninfected, submicroscopically infected, and microscopically infected/asymptomatic children living in high transmission setting. R_s_: Spearman correlation. Between group comparisons made with Wilcoxon ranksum test. Median indicated by black bar.

We have previously reported no significant relationship between concurrent parasitemia with either Vδ2^+^ T cell percentages or cytokine production from these cells following *in vitro* stimulation with P. *falciparum*–infected RBCs^9^. However, our earlier cohort had relatively few patients with microscopic parasitemia and a lack of data on sub-microscopic infection. In the present study, we found that children with microscopic parasitemia had significantly fewer circulating Vδ2^+^ T cells/μl than uninfected children or those with submicroscopic infection at the time of assay (Fig. 3a). We did not, however, observe a difference in Vδ2^+^ T cell counts between uninfected children and those with submicroscopic infection. We further examined the relationship between absolute Vδ2^+^ T cell counts and parasite densities among asymptomatic and symptomatic children with parasitemia. We found a significant inverse correlation between Vδ2^+^ T cell counts and parasite densities (Rs = −0.37, P = 0.001, Fig. 3b).

Similar results were obtained when evaluating the overall percentage of Vδ2^+^ T cells, and the percentage of Vδ2^+^ T cells that produced inflammatory cytokines (IFNg, TNF) following *in vitro* malaria antigen stimulation. Asymptomatic children with microscopic parasitemia had significantly lower overall Vδ2^+^ T cells percentages (Fig. 3c), and lower percentages of pro-inflammatory cytokine- producing Vδ2^+^ T cells (Fig. 3d), than uninfected children at the time of assay, but percentages were similar between uninfected children and those with submicroscopic infection. We also observed a significant inverse correlation between percentages of pro-inflammatory cytokine- producing Vδ2^+^ T cells and parasite densities among parasitemic children (Rs −0.24, P = 0.01). Together, these data reveal that concurrent microscopic parasitemia, and higher parasite densities, are associated with both lower counts and diminished pro-inflammatory cytokine production of Vδ2^+^ T cells.

### Vδ2^+^ T cells from children living in high transmission settings upregulate markers of exhaustion and replicative senescence which correlate with loss of effector functions

**Figure 4:**
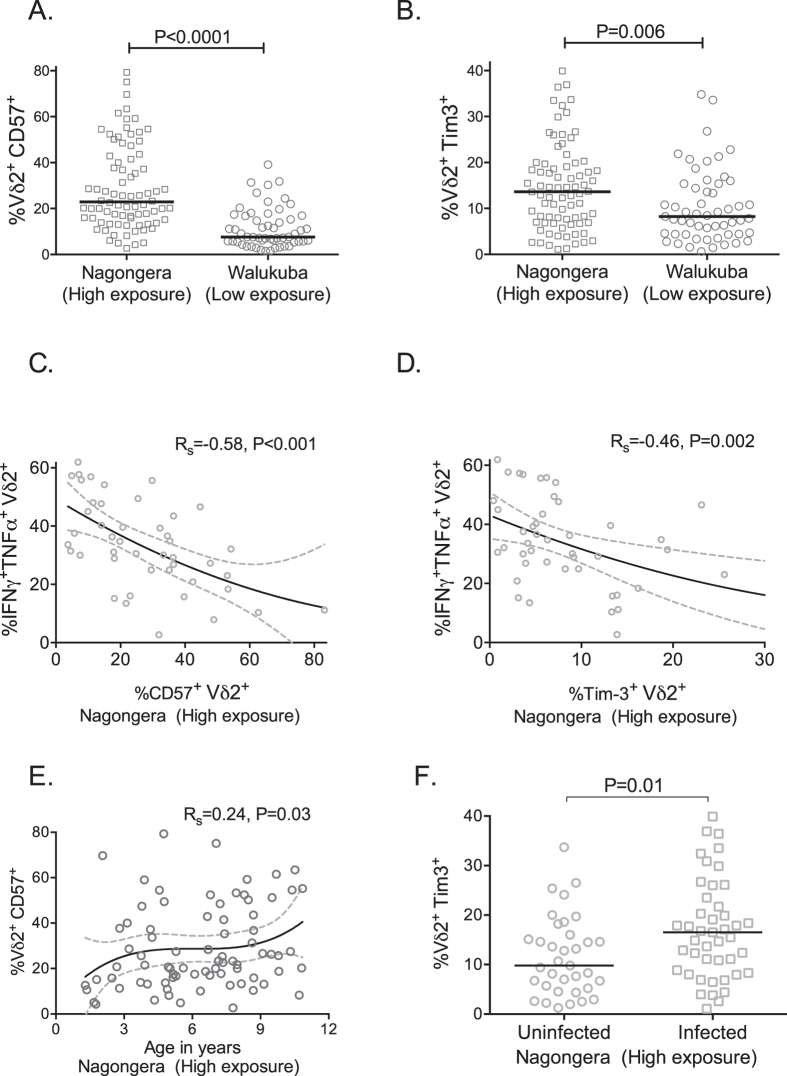
Vδ2^+^ T cell expression of markers of exhaustion and replicative senescence correlate with loss of effector functions. Vδ2 expression of CD57 (A) and Tim-3 (**B**) on freshly isolated PBMCs among asymptomatic children compared between children living in high (Nagongera, n = 80) vs low (Walukuba, n = 54) transmission settings. Both CD57 (**B**) and Tim-3 (**C**) expression associated with reduced Vδ2^+^ T cell cytokine production following *in vitro* stimulation. In high transmission setting, CD57 expression on Vδ2^+^ T cells associated with increasing age (**E**); Tim-3 expression associated with infection status at the time of measurement (**F**). R_s_: Spearman correlation. Between group comparisons made with Wilcoxon ranksum.

We have previously shown that repeated malaria exposure is associated with increased Vδ2 expression of several genes associated with immunomodulation^9^. These include the gene encoding CD57, a protein which has been associated with replicative senescence of CD8^+^ T cells in the setting of chronic antigen exposure^28^; and *HAVCR2*, which encodes the receptor Tim-3, a cell surface molecule that has been implicated in tolerance and exhaustion of Th1^29, 30^ and innate cells^31^. We assessed cell surface expression of CD57 and Tim-3 on Vδ2^+^ T cells and found expression of both markers to be significantly higher among children living in Nagongera in comparison to children living in Walukuba (Fig. 4a,b). Importantly, expression of both markers was associated with diminished Vδ2^+^ T cell cytokine production following stimulation (Fig. 4c,d). We also recently reported that CD16 expression identifies antigen-unresponsive Vδ2 T cells^23^. Consistent with these observations, we observed an inverse association between CD16 expression and Vδ2 production of IFNγ and TNFα (Rho = −0.62, P < 0.001).

CD57 expression on Vδ2^+^ T cells increased with age among children living in Nagongera (Rs = 0.24, P = 0.03, Fig. 4e), but not in children living in the low-endemicity setting of Walukuba (Rs 0.12, P = 0.38). In contrast, Tim-3 expression was not associated with age (Rs 0.11, P = 0.31), but was significantly higher among children with asymptomatic parasitemia (both submicroscopic and microscopic, P = 0.01, Fig. 4f) These data are consistent with the hypothesis that recurrent malaria leads to upregulation of multiple immunoregulatory pathways that dampen the immune response to malaria.

### Higher Vδ2^+^ T cell counts and cytokine production are associated with protection from P. falciparum infection, but also with a greater odds of symptoms if infected

Finally, to evaluate relationships between Vδ2^+^ T cell parameters and clinical immunity to malaria, we independently assessed associations with both the odds of subsequent infection and, secondarily, the odds of symptoms if infected. For these analyses, we measured associations with the infection status at the time of subsequent routine quarterly surveillance in the year following the assessment among children living in the high transmission setting of Nagongera.

#### Protection from P. falciparum infection

We found that higher frequencies of Vδ2^+^ T cells, as well as higher percentages of Vδ2^+^ T cells that produced IFNγ and TNF upon malaria antigen stimulation, were both associated with a significantly lower odds of subsequent *P. falciparum* infection in the subsequent year (Table 1). Children in the middle/highest strata for either of these parameters had a >50% reduced odds of subsequent infection compared to children in the lowest strata (P < 0.01 for all, Table 1). These results remained significant after adjustment for both age and household mosquito exposure. Similarly, higher absolute counts of Vδ2^+^ T cell were associated with a lower odds of infection (OR 0.36 per 10-fold increase in Vδ2^+^ T cell absolute counts, 95% CI 0.15–0.85, P = 0.02). We also evaluated associations with protection from higher density *P. falciparum* infection (i.e. the odds of sub-microscopic infection, given any infection). After adjusting for age, children in the middle/highest strata of Vδ2^+^ T cell IFNγ/TNF co-production had a significantly higher odds of submicroscopic infection if infected than children in the lowest strata (OR 1.46, 95% CI 1.01–2.11, P = 0.04). These results are consistent with the hypothesis that Vδ2^+^ T cells contribute to protection against infection, possibly by limiting parasite replication.

**Table 1:** Vδ2 frequency, functional strata and protection from P. falciparum infection in subsequent year

Predictor^a^	N (%)	Prevalence of any infection (n/N, %)	Unadjusted^b^			Adjusted^c^		
			OR	95% CI	p-value	aOR	95% CI	p-value
**Percentage Vδ2** ^**+**^ **among CD3** ^**+**^								
Low (0.06–0.38)	93 (35.1)	300/346, 88%	Ref	Ref	Ref	Ref	Ref	Ref
Middle (0.38–0.87)	92 (34.7)	258/340, 76%	0.44	0.26–0.75	0.002	0.49	0.30–0.82	0.007
High (0.88–8.7)	80 (30.2)	215/303, 71%	0.34	0.20–0.59	<0.001	0.35	0.21–0.59	<0.001
**Percentage IFNγ** ^**+**^ **/TNFα** ^**+**^ **Vδ2** ^**+**^								
Low (0.84–27.8)	95 (35.9)	298/350, 85%	Ref	Ref	Ref	Ref	Ref	Ref
Middle (28.2–40.3)	91 (34.3)	254/339, 75%	0.44	0.26–0.76	0.003	0.49	0.29–0.83	0.008
High (40.6–67.0)	79 (29.8)	221/300, 74%	0.41	0.24–0.72	0.002	0.47	0.27–0.81	0.007
**Age Category**								
0 - < 4	80 (30.2)	217/309, 70%	Ref	Ref	Ref	Ref	Ref	Ref
≥4 - < 7	87 (32.8)	272/336, 81%	2.00	1.19–3.38	0.009	1.64	0.99–2.73	0.055
≥7 - < 11	98 (37.0)	284/344, 83%	2.08	1.23–3.51	0.006	1.79	1.08–2.98	0.025
**Mosquito Exposure Rate**								
1 - < 8	18 (6.8)	43/66, 65%	Ref	Ref	Ref	Ref	Ref	Ref
≥8 - < 40	193 (72.8)	564/724, 78%	2.12	0.86–5.24	0.102	2.40	1.04–5.56	0.04
≥40–80	40 (15.1)	122/150, 81%	2.67	0.93–7.64	0.067	3.58	1.34–9.53	0.011
≥80	14 (5.3)	44/49, 90%	6.22	1.26–30.63	0.025	8.60	1.96–37.64	0.004

OR: odds ratio; aOR: adjusted odds ratio

^a^Includes n = 265.

^b^For analysis, all routine visits were considered. Odds of any infection at routine visits includes both submicroscopic and microscopic infections.

^c^Adjusted for age category and mosquito exposure rate.

#### Protection from symptoms once infected with P. falciparum

**Figure 5:**
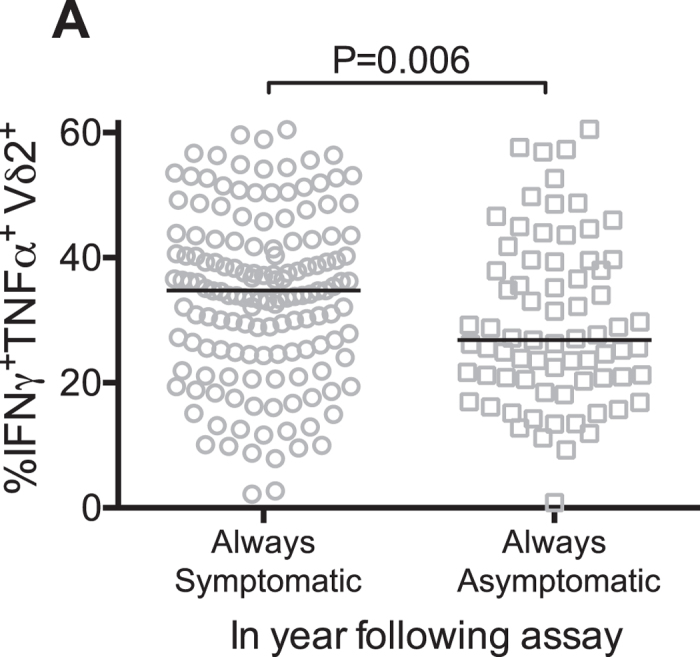
Children with fewer pro-inflammatory cytokine-producing Vδ2^+^ T cells are more likely to have asymptomatic infection. Percentage of Vδ2^+^ T cells producing cytokines following *P*. *falciparum* stimulation in children with only symptomatic vs. only asymptomatic infection in year following assay.

We next analyzed associations between Vδ2^+^ T cell parameters and the prospective odds of having symptoms given microscopic infection. Higher percentages of Vδ2^+^ T cells producing inflammatory cytokines IFNγ and TNF following *in vitro* stimulation was associated with an *increased* odds of having symptoms given *P. falciparum* infection. Children in the middle and highest strata of Vδ2^+^ T cell cytokine production had a >3 times higher odds of symptoms given patent blood smear infection in the subsequent year compared to children in the lowest strata (P = 0.004, Table 2). These results remained significant after adjusting for age (Table 2). Consistent with these findings, children who only experienced asymptomatic infection in the subsequent year had significantly lower percentages of IFNγ/TNF-producing Vδ2^+^ T cells than children whose infections were all symptomatic (P = 0.006, Fig. 5). The frequencies and absolute counts of Vδ2^+^ T cell were not associated with the risk of symptoms once infected. Together, these results suggest that, among children living in a high transmission setting, the frequency and function of Vδ2^+^ T cells are associated with protection from subsequent infection, independent of age and mosquito exposure. However, once microscopic infection is established, pro-inflammatory cytokine production from these cells are associated with a greater likelihood of symptoms.

**Table 2:** Vδ2 functional strata and odds of symptoms if P. falciparum infected by microscopy in subsequent year

Predictor	N(%)	Symptoms if infected (n/N, %)	Unadjusted			Adjusted^c^		
			OR	95% CI	p-value	aOR	95% CI	p-value
**Percentage IFNγ** ^**+**^ **/TNFα** ^**+**^ **Vδ2** ^**+c**^								
Low (0.84–27.8)	95 (35.9)	73/189, 38%	Ref	Ref	Ref	Ref	Ref	Ref
Middle (28.2–40.3)	91 (34.3)	91/148, 61%	3.69	1.77–7.69	<0.001	2.73	1.39–5.36	0.003
High (40.6–67.0)	79 (29.8)	69/122, 57%	3.08	1.42–6.67	0.004	2.27	1.11–4.64	0.025
**Age Category**								
0 - < 4	80 (30.2)	97/138, 70%	Ref	Ref	Ref	Ref	Ref	Ref
≥4 - < 7	87 (32.8)	85/176, 48%	0.30	0.15–0.60	0.001	0.35	0.18–0.70	0.003
≥7 - < 11	98 (37.0)	51/145, 35%	0.14	0.07–0.30	<0.001	0.17	0.16–0.47	<0.001

OR: odds ratio; aOR: adjusted odds ratio.

^a^Includes n = 225.

^b^For analysis, only routine quarterly visits with microscopic infection were considered. Symptomatic malaria considered if blood smear positive and fever at visit, or within 21 days prior and 7 days following visit.

^c^Adjusted for age category.

## Discussion

In this study, we extend our prior work showing that repeated malaria in children is associated with relative loss and *in vitro* dysfunction of Vδ2^+^ T cells,^9, 23^ and identify a potentially important role for Vδ2^+^ T cells in clinical immunity to malaria. We observed that, in a high transmission setting, increasing age – a proxy for cumulative malaria exposure – was associated with diminished *in vivo* Vδ2^+^ T cell expansion following malaria and loss of absolute numbers of Vδ2^+^ T cells, consistent with the hypothesis that repeated malaria is associated with both *in vitro* and *in vivo* dysfunction of these cells. Microscopic parasitemia and Vδ2 expression of Tim-3 and CD57 – two immunoregulatory markers found to be increased among children highly exposed to malaria - were associated with diminished Vδ2^+^ T cell pro-inflammatory cytokine production. Finally, by leveraging longitudinal follow-up among a large cohort of children of varying ages, we observed that higher Vδ2^+^ T cell counts and cytokine production were associated with protection from subsequent *Pf* infection among children living in a high transmission setting. However, cytokine production from these cells was also associated with an increased probability of symptoms once infected. Together, these results support the hypothesis that loss and dysfunction of Vδ2^+^ T cells may represent a disease tolerance mechanism that facilitates the development of clinical immunity to malaria among children.

Prior studies conducted among malaria-naïve individuals and adults have found frequencies of γδ T cells to reach as high as 30% of circulating T cells following a symptomatic malaria episode^24^. We found that Vδ2^+^ T cells expand following symptomatic malaria among children living in an endemic setting, but that this expansion occurs primarily among young children who have had less prior malaria exposure and is significantly diminished among older children. As older children living in high transmission settings have experienced significantly more cumulative episodes of malaria during their lifetime then younger children, we speculate that the diminished expansion observed may be a result of repeated malaria infection. This is further supported by our observation that repeated malaria results in both decreased *in vitro* proliferation and cytokine production of Vδ2^+^ cells in response to malaria antigen stimulation^9, 23^. Furthermore, we have shown that repeated malaria exposure is associated with increased expression of immunoregulatory markers, including Tim-3^9^, whose expression is associated with diminished *in vitro* function of these cells, consistent with observations from other groups^32^.

We further observed an age-associated decrease in Vδ2^+^ T cell absolute counts among children living in a high transmission setting, but not in a low transmission setting. Two other reports in non-malaria endemic settings evaluated Vδ2^+^ T cells from birth through adulthood, and both reported an expansion of this subset with increasing age^26, 27^. Both of these studies also described increased expression of CD45RO among this cellular subset, a marker suggesting antigen experience. Given that Vδ2^+^ T cells respond to small molecular weight phosphoantigens generated by many bacterial, mycobacterial, and parasitic organisms^33–35^, one may speculate that occasional exposure to low-density infections early in life may drive proliferation and positive selection of these cells. However, our observations suggest that in areas where malaria is endemic, continuous activation may lead to loss of Vδ2^+^ T cells in the peripheral circulation.

In the present study, we found that higher Vδ2^+^ T cell absolute counts, frequencies, and cytokine production were associated with protection against subsequent *Pf* infection, and that these results remained significant after adjusting for heterogenous exposure to malaria-infected mosquitoes. Furthermore, higher frequencies of malaria-responsive Vδ2^+^ T cells producing inflammatory cytokines were associated with a greater likelihood that subsequent infections would be below the level of detection by microscopy. Together, these results suggest that Vδ2^+^ T cells may indeed play a role in limiting parasite replication *in vivo* ^19^. These results are consistent with studies from school-aged children in Papua New Guinea which found that γδ production of IFNγ and/or TNF^20, 36^ were associated with protection from clinical malaria, and a recent study from individuals receiving an experimental attenuated sporozoite vaccine which found that higher frequencies of Vδ2^+^ T cells correlated with protection from subsequent *Pf* challenge^21^. In our prior studies, we did not observe significant associations between Vδ2^+^ T cell parameters and protection from *Pf* infection^9^, though these studies were performed in younger children (<5 yrs of age) and did not assess for submicroscopic infection.

Though Vδ2^+^ T cells may possess effector functions that that can limit parasite replication, we found that higher frequencies of cytokine-producing Vδ2^+^ T cells were associated with a higher probability of symptoms given infection. This observation is consistent with published data suggesting that cytokine production from these and other cells may be responsible, in part, for mediating clinical symptoms^9, 22^. Taken together, our observations are consistent with the hypothesis that repeated malaria may drive a disease tolerance pathway among Vδ2^+^ T cells that attempts to reduce the negative impact of infection on host fitness^37^. Mechanisms of malaria-induced Vδ2^+^ T cell dysfunction remain to be elucidated, but may include signaling through Tim-3^32^ and/or other immunoregulatory pathways, and/or changes to the epigenetic landscape of these cells^38^, similar to what has been described in several innate cellular populations in response to infection^39, 40^.

Given the intrinsic reactivity of Vδ2 T cells to *P. falciparum* and their direct anti-merozoite effects *in vitro*, we speculate that these cells may play a beneficial role as ready-made effectors during primary acute malaria infection of infants and young children, before the development of an effective adaptive immune response^41^. Although the progressive loss and dysfunction of these cells with repeated exposure is associated with reduced symptoms, it may conceivably interfere with effective clearance of the infection in childhood, contributing to rising parasite prevalence with increasing age in high transmission settings^5, 8, 42^. Although individuals rarely if ever develop sterilizing immunity that prevents any infection, control of blood-stage parasite density improves with increasing age, which we speculate may be due to the eventual development of adaptive immune responses, including antibodies, that develop with repeated exposure. Furthermore, it is possible that as Vδ2 T cells downregulate production of pro-inflammatory cytokines, they acquire additional effector functions, perhaps mediated by CD16 (*FcgRIII*), which is upregulated following repeated exposure to malaria^9, 23^. In other contexts, CD16^+^ Vδ2 T cells have been reported to mediate phagocytosis^43^; hence it is possible that in older children with chronic malaria exposure, Vδ2 T cells may work in concert with antimalarial antibodies to restrict parasite burden.

There were several limitations to this study. Although we observed age-related differences in Vδ2^+^ T cells that parallel the development of clinical immunity to malaria, we cannot determine whether these changes are causally responsible for clinical immunity. Many immune effector populations are regulated in concert, any of which may contribute to this process. Furthermore, the age-associated loss and dysfunction of Vδ2^+^ T cells observed in peripheral blood in heavily malaria-exposed children could potentially reflect redistribution to the spleen, liver, or other tissue sites in response to malaria infections. Finally, given the cross-sectional nature of the phenotypic and functional assessments described, associations with protection from infection and symptoms given infection should be interpreted cautiously, and require validation in other cohorts and exploration in model systems.

In conclusion, our data support a growing body of studies which suggest that Vδ2^+^ T cells may play an important role in protection from *Pf* infection^20, 21, 36^. However, clinical immunity to malaria may be mediated by disease tolerance mechanisms that down-modulate pro-inflammatory responses from Vδ2^+^ and/or other cellular subsets^9–11, 37^ and may interfere with the ability of hosts to clear and/or prevent re-infection. Although clinical immunity may protect children from malaria-associated morbidity and mortality, asymptomatic infection is an important driver of *Pf* transmission^6, 7^. Understanding the mechanisms and determinants of clinical immunity thus remains critically important for the success of malaria elimination strategies and for the development of novel vaccination strategies.

## Methods

### Ethical approval

Written informed consent was obtained from the parent or guardian of all study participants. The study protocols were approved by the Uganda National Council of Science and Technology (HS 1019), the Makerere University School of Medicine Research and Ethics Committee (Rec No. 2011–167), and the University of California, San Francisco Committee on Human Research (11–05995). All research was performed in accordance with the Declaration of Helsinki and Good Clinical Practice.

### Study participants and clinical procedures

Samples were obtained from children enrolled in the East African International Centers of Excellence in Malaria Research cohorts. These participants live in 100 randomly selected households from each of two study sites with different transmission intensities: 1) Nagongera sub-county in Tororo district, a setting with holoendemic malaria transmission and an estimated annual entomological inoculation rate (aEIR) of 310 bites per person year, and 2) Walukuba sub-county in Jinja district, a lower transmission setting with an aEIR of 2.8 infectious bites per year^44^. In all households, all eligible children aged 6 months to 10 years were enrolled into the study beginning in August of 2011. The cohorts are dynamic, with children exiting the study at age 11, and new children born into study households enrolled at 6 months of age.

Upon enrollment all study participants were given an insecticide treated bed net and followed for all medical care at a dedicated study clinic. All participants were reimbursed for travel to and from clinic, and participants agreed to avoid all antimalarial medications administered outside the study. Children who presented with a fever (tympanic temperature >38.0 °C) or history of fever in the previous 24 hours had blood obtained by finger prick for a thick smear. If the thick smear was positive for *Plasmodium* parasites, the patient was diagnosed with malaria regardless of parasite density, and treated with artemether-lumefantrine. Routine assessments were performed in the study clinic every three months, including blood smears and dry blood spots to detect for parasite infection. Negative blood smears obtained at routine assessments were tested for the presence of submicroscopic malaria parasites using loop-mediated isothermal amplification (LAMP)^45^.

### Entomologic surveys

Entomological surveys were conducted monthly beginning in October 2011^44^. Each month, miniature Centers for Disease Control and Prevention light traps (Model 512; John W. Hock Company, Gainesville, FL) were positioned with the light 1 meter above the floor at the foot end of the bed where a cohort study participant slept. Traps were set at 7.00 PM and collected at 07.00 AM the following morning by field workers. A mosquito exposure rate was calculated for each individual based on the number of female Anopheles mosquitoes captured during the 12 months following the blood draw/number of house nights of collection^44^. Mosquito exposure rate was used in analysis as a categorical variable (1- < 8, 8- < 40, 40- < 80 and ≥80 mosquitos/household/day).

### Acute and convalescent malaria absolute count measurements

Repeated assessments of T cell absolute counts were performed at the time of symptomatic malaria and 3,6, and 9 weeks following the malaria episode, in a subset of children in the Nagongera cohort (n = 43). Children were enrolled in this “symptomatic malaria cohort” if they met the following criteria: 1) duration of fever <48hrs; 2) *P. falciparum* infection with a parasite density >5,000/uL; 3) absence of complicated malaria; 4) no clinical suspicion of concurrent non-malarial illness; 5) no malaria in the prior month. Convenience sampling was used to select study participants to ensure that similar numbers of children in each of 3 age groups (0.5 - < 4 yrs, 4 - < 7 yrs, and 7- < 11 yrs) were included. At the time of each blood draw, 3 to 5 mls of blood were obtained in acid citrate dextrose tubes. 25 μl of fresh whole blood was stained with CD3 PerCP, CD4 FITC, Vδ2 PE, and Vγ9 APC (all Biolegend) for 20 minutes in a Trucount Tubes (Becton Dickinson) and then lysed and permeabilized with BD FACS Lyse buffer prior to acquisition on an Accuri C6 Cytometer (BD Biosciences).

### Assessments in asymptomatic children at the time of routine assessments

Vδ2 phenotypic, absolute count, and functional assessments were obtained using convenience sampling at the time of routine assessments in age-matched children enrolled in both the high (Nagongera, n = 283) and lower transmission (Walukuba, n = 94) cohorts. Not all assessments were performed in all individuals due to sample availability. Measurements of cell surface phenotype and absolute counts were made utilizing fresh samples obtained at routine visits occurring between February of 2014 and July of 2015. Measurements of cytokine production following *in vitro* stimulation were made using cryopreserved specimens collected between February and August of 2013 at a scheduled “immunology visit.” For this “immunology visit”, children were bled if they were currently afebrile and did not have a recorded episode of malaria in the last 7 days. For this “immunology” visit, negative blood smears were tested for the presence of submicroscopic malaria parasites using PCR.

#### Cell surface phenotype

At the time of routine visit, 3 to 5 mls of blood were obtained in acid citrate dextrose tubes. Absolute count measurements were performed on 25 μl of fresh whole blood as above. Peripheral blood mononuclear cells (PBMC) were then isolated by density gradient centrifugation (Ficoll-Histopaque; GE Life Sciences) and counted. 1 × 10^6 freshly isolated PBMC were stained for 30 minutes with the following cell-surface antibodies: from BD Pharmingen, anti-CD8-APC-H7 (SK1); from Biolegend, anti-CD3-Pacific Blue (SK7), anti-Vδ2^—^PerCP (B6), anti-Vγ9 APC (B3), and anti-CD57 FITC (HCD57) and from R&D, anti-Tim-3-PE (344823), fixed with 1% paraformaldehyde, and acquired on a FACS Canto flow cytometer (BD Biosciences) with FACSDiva software.

#### Vδ2 in vitro cytokine production

At the time of the “immunology visit,” 6 to10 mls of blood were obtained in acid citrate dextrose tubes. Peripheral blood mononuclear cells (PBMC) were isolated by density gradient centrifugation (Ficoll-Histopaque; GE Life Sciences), counted, and cryopreserved in liquid nitrogen prior to use. Analysis of Vδ2 T cell responses to P. *falciparum* infected red blood cell (RBC) stimulation via intracellular cytokine staining was performed as previously described^9^. Briefly, thawed PBMCs were rested overnight in 10% fetal bovine serum (Gibco) and counted before stimulation with P. falciparum infected RBCs or uninfected RBCs. A total of 10^6^ PBMCs were stimulated with intact purified trophozoite/schizont-stage P. falciparum (clone 3D7)–infected RBCs or uninfected RBCs at an effector to target ratio of 1:2. Parasite cultures were routinely confirmed to be mycoplasma negative. Following 6 hours of stimulation, Brefeldin A and monensin (BD Pharmingen) were added (10 μg/mL). At 24 hours, cells were washed, and surface and intracellular staining was performed with the following antibodies: from BD Pharmingen, anti-CD3-PerCP (SK7), anti-CD8-APC-H7 (SK1), anti-IFNγ- PE-Cy7 (B27), and anti-TNFα- FITC (6401.1111); from Biolegend, anti-CD4-BV650 (OKT4), anti-CD14-Alexa700 (M5E2), anti-CD19-Alexa700 (HIB19); from Miltenyi anti- Vδ2^—^APC (123R3); and from Invitrogen, Live/Dead aqua amine. Additional experiments utilized anti-Tim-3 PE (R&D Biosciences, clone 344823) and anti-CD57 BV 421 (Biolegend, Clone HCD57). Samples were acquired on a BD LSR2 flow cytometer with FACSDiva software.

### Flow cytometric data analysis

Flow cytometry data were analyzed using FlowJo software (Tree Star, San Carlos, California). Color compensation was performed using single-color cell controls or beads stained for each fluorochrome. Absolute cell counts were calculated using the manufacturer recommendations (BD Biosciences). For surface phenotype and functional assays, cells were gated as lymphocytes/singlets/CD14^−^CD19^−^Aqua^−^/CD3^+^/Vδ2^+^. For cell surface phenotypes, gates were set using fluourescence-minus one controls. Cytokine production was gated as the percentage of Vδ2^+^ T cells producing IFNγ and TNFα. To calculate frequencies of P. *falciparum*–specific T cells, background responses to uninfected RBCs were subtracted from each subset. Thresholds for cell surface phenotype were set based on fluorescence-minus-one controls.

### Statistical data analysis

All statistical analyses were performed using Prism 6.0 (GraphPad) and/or STATA version 14 (College Station).

#### Acute and convalescent malaria

Comparisons of absolute counts between timepoints were made using the Wilcoxon matched pairs signed-rank test. Associations between age strata and Vδ2 T cell absolute counts at 0, 3, 6, and 9 weeks following acute malaria were measured using generalized estimating equations regression analysis with robust standard errors to account for repeated measures in the same child, after adjustment for day 0 parasite density. These models were assessed for significant interaction between age strata and weeks following the acute malaria episode.

#### Asymptomatic children

Comparisons of immune parameters between groups were made using the Wilcoxon rank sum test. Continuous variables were compared using Spearman correlation. Associations between Vδ2 T cell responses and the subsequent odds of infection or symptoms when infected were assessed in the year following the assessment. For these analyses, only the infection status at the time of routine quarterly surveillance was considered. At each routine assessment, children were divided into one of four mutually exclusive categories: 1) Symptomatic malaria, with a window of 21 days prior to and 7 days following the routine visit to ensure capture of malaria episodes that were recently treated or infections that soon became symptomatic; 2) Asymptomatic, microscopic (blood smear+) infection; 3) Asymptomatic, submicroscopic (LAMP+) infection; or 4) No evidence of parasite infection. Associations between Vδ2 T cell responses and the prospective odds of any infection (including submicroscopic and microscopic infections), sub-microscopic infection (given infection), or symptoms when infected (detected by microscopy) were calculated using multilevel mixed-effects logistic regression. This analysis accounted for repeated measures within individuals and was clustered on household to account for multiple children living in the same household. In multivariate analysis, odds ratios for infection risk were adjusted for age (categorical) and mosquito exposure (categorical); odds ratios for symptoms when infected were adjusted for age. In all models, non-normal variables were log-transformed where appropriate. In all analyses, a 2-tailed P value < 0.05 was considered to be statistically significant.

## Electronic supplementary material

Supplementary Figures
