# Supplementary material for: Vδ2+ T cell response to malaria correlates with protection from infection but is attenuated with repeated exposure
Source: Sci Rep. 2017 Sep 13;7:11487. doi: 10.1038/s41598-017-10624-3 (PMC5597587; doi:10.1038/s41598-017-10624-3)
Supplement: Supplementary file 1 — Supplementary Figures [file 41598_2017_10624_MOESM1_ESM.pdf]

Vδ2+ T cell response to malaria correlates with protection from infection but is attenuated with repeated exposure

Prasanna Jagannathan,[1,2*] Fredrick Lutwama,[3,4+] , Michelle J. Boyle,[2,5+], Felistas Nankya,[6] Lila A. Farrington,[2] Tara I.McIntyre,[2] Katherine Bowen,[2] Kate Naluwu,[6] Mayimuna Nalubega,[6] Kenneth Musinguzi,[6] Esther Sikyomu,[6] Rachel Budker,[2] Agaba Katureebe,[6] Rek John,[6] Bryan Greenhouse,[2] Grant Dorsey,[2] Moses R. Kamya,[4] Margaret E.Feeney[2,7*].

[1]Department of Medicine, Stanford University, Stanford, CA, USA

[2]Department of Medicine, University of California San Francisco, San Francisco, CA, USA

[3]Infectious Diseases Institute, Kampala, Uganda

[4]Makerere University College of Health Sciences, Kampala, Uganda

[5]Burnet Institute, Disease Elimination (Malaria), Melbourne, Australia;

[6]Infectious Diseases Research Collaboration, Kampala, Uganda

[7]Department of Pediatrics, University of California San Francisco, San Francisco, CA, USA

[+]These authors contributed equally

*prasj@stanford.edu, margaret.feeney@ucsf.edu

Supplementary Figures

Supplementary Figure S1

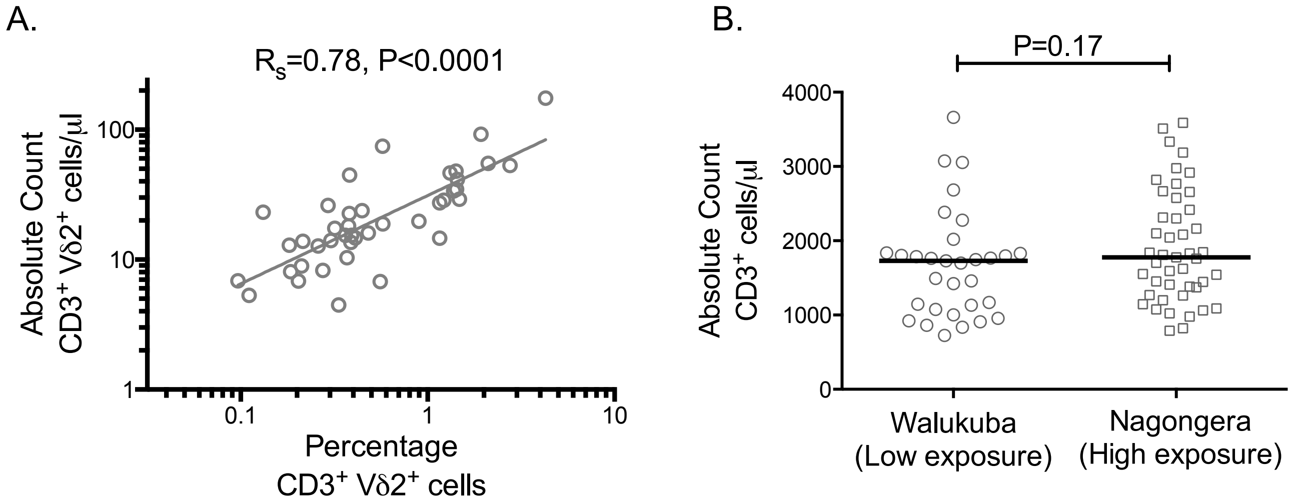

Supplementary Figure S1. A. Correlation between absolute count CD3+ Vδ2+ cells/µl and Vδ2+ T cell percentages. Rs: Spearman Rho. B. Absolute CD3+ cells/µl among asymptomatic children aged 6 months to 11 years in high (Nagongera) vs low (Walukuba) transmission setting at the time of routine assesments, Wilcoxon Rank Sum.

Supplementary Figure S2

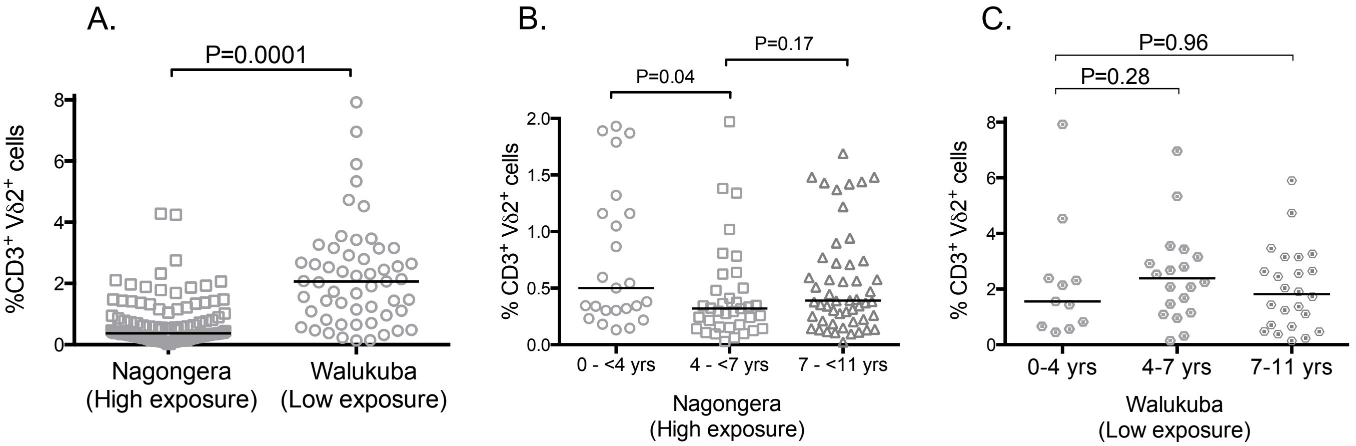

Supplementary Figure S2. Vδ2+ T cell percentages decline with increasing age among children living in high transmission setting. A. Percentage CD3+ Vδ2+ cells among asymptomatic children aged 6 months to 11 years in high (Nagongera) vs low (Walukuba) transmission setting at the time of routine assesments. B. Percentage CD3+ Vδ2+ cells by age categories in Nagongera. B. Percentage CD3+ Vδ2+ cells by age categories in Walukuba. Wilcoxon Rank Sum.
